# The Neuroprotective Effect of the X Protein of Orthobornavirus Bornaense Type 1 in Amyotrophic Lateral Sclerosis

**DOI:** 10.3390/ijms252312789

**Published:** 2024-11-28

**Authors:** Jeflie Tournezy, Claire Léger, Bernard Klonjkowski, Daniel Gonzalez-Dunia, Marion Szelechowski, André Garenne, Stéphane Mathis, Stéphanie Chevallier, Gwendal Le Masson

**Affiliations:** 1 Neurocentre Magendie INSERM U1215, Université de Bordeaux, 33000 Bordeaux, France; jeflie.tournezy@inserm.fr (J.T.); claire.leger@inserm.fr (C.L.); gwendal.le-masson@chu.fr (G.L.M.); 2 UMR 1161 Virologie, INRA, ANSES, Ecole Nationale Vétérinaire d’Alfort, 94700 Maisons-Alfort, France; bernard.klonjkowski@vet-alfort.fr; 3 Infinity (Toulouse Institute for Infectious and Inflammatory Diseases), INSERM, CNRS, Université de Toulouse, UPS, 31024 Toulouse, France; daniel.dunia@inserm.fr (D.G.-D.); marion.szelechowski@inserm.fr (M.S.); 4 IMS Laboratory, UMR5218, CNRS, Bordeaux University, 33400 Talence, France; andre.garenne@u-bordeaux.fr; 5 Nerve-Muscle Unit, ALS Center, Department of Neurology, University Hospital (CHU) of Bordeaux (Pellegrin Hospital), 33000 Bordeaux, France

**Keywords:** ALS, mitochondria, BoDV-1, X protein, SOD1^G93A^ mice

## Abstract

In amyotrophic lateral sclerosis (ALS), early mitochondrial dysfunction may contribute to progressive motor neuron loss. Remarkably, the ectopic expression of the Orthobornavirus bornaense type 1 (BoDV-1) X protein in mitochondria blocks apoptosis and protects neurons from degeneration. Therefore, this study examines the neuroprotective effects of X protein in an ALS mouse model. We first tested in vitro the effect of the X-derived peptide (PX3) on motoneurons primary cultures of SOD1^G93A^ mice. The total intracellular adenosine triphosphate (ATP) content was measured after incubation of the peptide. We next tested in vivo the intramuscular injection of X protein using a canine viral vector (CAV2-X) and PX3 intranasal administrations in *SOD1^G93A^* mice. Disease onset and progression were assessed through rotarod performance, functional motor unit analysis via electrophysiology, and motor neuron survival by immunohistochemistry. The results showed that in vitro PX3 restored the ATP level in SOD1^G93A^ motor neurons. In vivo, treated mice demonstrated better motor performance, preserved motor units, and higher motor neuron survival. Although life expectancy was not extended in this severe mouse model of motor neuron degeneration, the present findings clearly demonstrate the neuroprotective potential of X protein in a model of ALS. We are convinced that further studies may improve the therapeutic impact of X protein with optimized administration methods.

## 1. Introduction

Amyotrophic lateral sclerosis (ALS) is a fatal, non-curable neurodegenerative disease for which the etiology remains unclear. ALS is currently recognized as a multisystem neurodegenerative disorder with disease heterogeneity at the clinical, genetic, and neuropathological levels [1]. Despite the fact that most ALS cases are sporadic (sALS), approximately 10% are familial (fALS) and associated with several genetic mutations in different genes such as *C9orf72*, *FUS*, *TARDBP*, and *SOD1* [2,3]. The first ALS-associated gene, superoxide dismutase-1 (*SOD1*) [4], was identified in 1993 and accounts for 12–20% of familial and 1–2% of sporadic ALS cases [4,5]. Over 185 disease-associated variations in *SOD1* have now been identified, distributed throughout the gene [6]. Phenotype, disease duration, and severity can differ significantly depending on the specific variants involved [7]. The discovery of the G93A mutation led to the development of murine models, which have been extensively studied and used in preclinical research. In this model, transgenic expression of the human SOD1 protein results in a neurodegenerative, paralytic process in mice that mimics many aspects of human ALS [8]. In patients with ALS, due to the SOD1 mutation, lower limb onset, and predominant lower motor neuron involvement are relatively common [6]. Moreover, patients with the G93A variant typically exhibit rapid disease progression and shorter survival. In *SOD1^G93A^* mice, a rapid disease progression has also been observed, largely depending on the number of mSOD1 copies [9]. These mice also show lower limb onset with progressive paralysis of the hindlimbs, coinciding with neurodegeneration of lumbar motor neurons [10]. Thus, although this model represents only a portion of the mutations involved in ALS, its study has contributed to the development of potential treatments.

Both in sALS and fALS patients as well as in animal models, among the pathophysiological hallmarks observed, mitochondrial dysfunctions appear to be one of the earliest events and are likely to be involved in the progressive loss of motoneurons (MNs) [11,12,13]. Notably, all of the different mouse models exhibit mitochondrial abnormalities despite their different mutations, suggesting an important role of these organelles in the pathophysiology of the disease. These organelles not only act as cellular powerhouses of neurons via oxidative phosphorylation but also integrate numerous cellular signaling pathways, including apoptosis, and produce toxic reactive oxygen species (ROS). We have already shown that mitochondrial dysfunction in MNs triggers a shortage in adenosine triphosphate (ATP) production that makes MNs unable to cope with any increase in their energy demand. As suggested previously [14], this bioenergetics crisis can induce apoptotic cell death.

Cell death is one of the most common antiviral consequences. To optimize their survival, many viruses hijack apoptosis, mainly by expressing proteins that target apoptotic pathways at the mitochondrial level. Among them, the Orthobornavirus bornaense type 1 (BoDV-1) can persist in the neurons of infected animal brains through the expression of a small 10 kDa protein called the ‘X protein’ [15]. When targeted to mitochondria, this protein inhibits the induction of apoptosis in infected cells, thus promoting noncytolytic viral persistence [16]. Moreover, it was recently demonstrated that the mitochondrial localization of X protein (or a peptide derived from this protein, called PX3) protects neurons from the earliest stages of degeneration in a model of Parkinson’s disease [15,17]. It was shown that mitochondrial targeting of the X protein preserves mitochondrial function, inhibiting the induction of oxidative stress and enhanced mitochondrial filamentation. Taken together, the results show that the neuroprotective effect of this protein is largely dependent on its ability to effectively target the mitochondria.

In this study, we aimed to examine the putative *neuroprotective* role of X protein in a mouse model of ALS (*SOD1^G93A^* mice). First, we took advantage of the property of X protein to rescue ATP production in culture motoneurons of *SOD1^G93A^* mice. Second, different strategies of X protein administration were used to evaluate the effects of a generalized administration of this protein. The delivery of the protein was performed either through intranasal instillation of the X-derived peptide (PX3) alone or by intramuscular injection of a canine adenovirus vector (CAV2) expressing the full-length X protein combined with intranasal instillation of PX3.

## 2. Results

### 2.1. Effect of the X-Derived Peptide PX3 In Vitro

**Figure 1:**
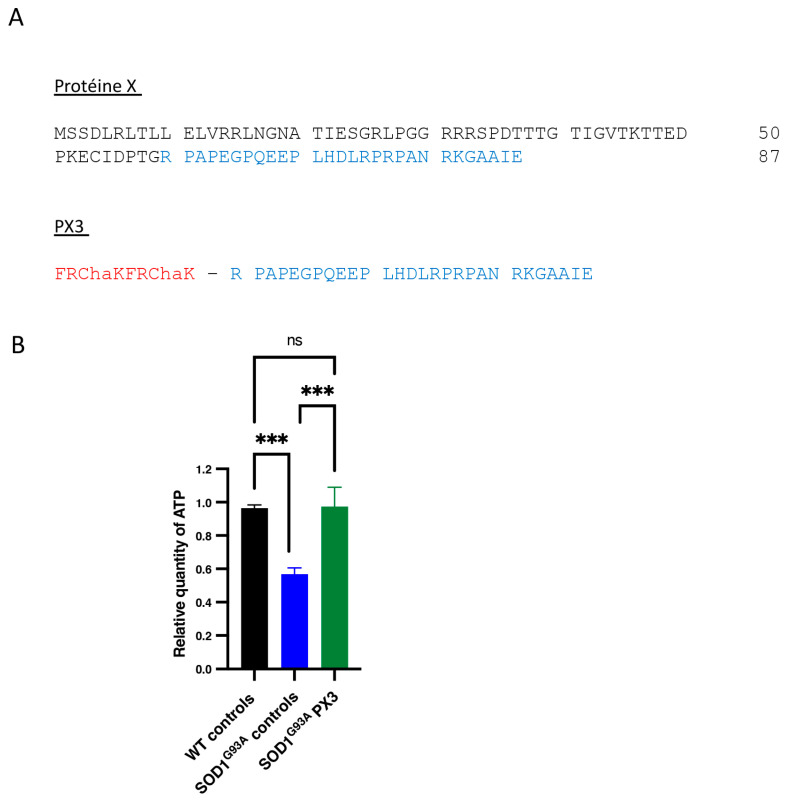
**X-derived peptide** PX3 restores ATP levels in primary cultures of *SOD1^G93A^* motor neurons. (**A**) Amino acid sequences of X protein and PX3 peptide. The X protein consists of 87 amino acids. The PX3 peptide corresponds to the carboxy-terminal 29 amino acids of X protein (highlighted in blue) to which a cell-permeable sequence (MPP, highlighted in red) is added. (**B**) The histogram bars represent the means of the relative amounts of ATP obtained in three independent cultures for each condition (WT controls, *n* = 17; SOD1^G93A^ controls, *n* = 11; SOD1^G93A^ PX3, *n* = 9). Error bars represent SEM. The data were analyzed by a Kruskal–Wallis test followed by a Conover test. ns: not significant; *** *p* < 0.001.

Mitochondrial dysfunction is a common feature of many neurodegenerative disorders, including ALS. It was shown that in *SOD1^G93A^* mice, oxidative phosphorylation is altered and mitochondrial respiration is reduced, leading to decreased ATP production [13,18]. In the present study, the effect of the X-derived peptide PX3 on primary cultures of MNs was tested. The PX3 peptide corresponds to the C-terminal region of the X protein spanning amino acids 59 to 87 (Figure 1A). Additionally, PX3 includes an MPP sequence (F-R-Cha-K-F-R-Cha-K, where Cha represents cyclohexylalanine), which enables efficient translocation across the plasma membrane and ensures high-specificity mitochondrial targeting [13].

Embryonic MNs of SOD1^G93A^ mice were obtained from the ventral spinal cord of embryonic mice (E12.5). As previously described, the amounts of ATP were greatly reduced in the cells at this very early stage. However, the addition of PX3 to the culture medium for 2 days was sufficient to completely restore ATP concentrations similar to those of WT MNs (Figure 1B).

### 2.2. Administration of X Protein and PX3 Peptide in SOD1^G93A^ Mice

**Figure 2:**
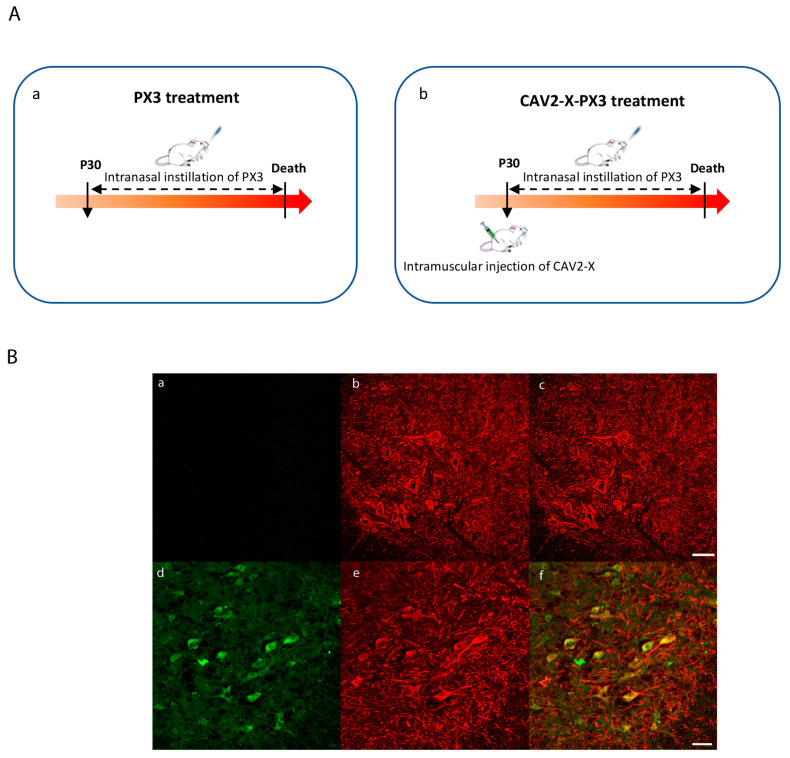
Administration of the CAV2-X and PX3 peptides (**A**) and expression of the X protein in SOD1^G93A^ mice (**B**). (**A**): Animals received intranasal instillations of the PX3 peptide alone (**a**) or as part of a combination therapy that included a viral vector-expressing protein X (CAV2-X) together with intranasal PX3 instillations (**b**). A single injection of CAV2-X was carried out bilaterally in the *triceps surae* (*Ts*) muscles at the age of 30 days in wild-type and *SOD1^G93A^* mice. Intranasal instillations of the PX3 peptide were performed three times per week from the age of 30 days until the death of the animal. (**B**): Spinal cord sections of control mice (**a**–**c**) and mice treated with CAV2-X-PX3 (**d**–**f**). (**a**,**d**) Ventral horn immunolabeled by an antibody directed against the X protein (**b**,**e**) Motor neurons immunolabeled with an anti-SMI32 antibody. (**c**,**f**) Superposition of (**a**,**b**) and of (**d**,**e**). In the later image (**f**), expression of the X protein in motor neurons is observed. Scale bar: 50 µm.

To investigate the potential therapeutic effect of X protein in vivo, we treated *SOD1^G93A^* mice with the derived peptide PX3 alone (PX3 treatment, Figure 2(Aa)) or as part of a combination therapy that included a viral vector expressing protein X (CAV2-X) together with intranasal PX3 instillations (CAV2-X-PX3 treatment, Figure 2(Ab)). The PX3 peptide was delivered by intranasal instillations while full-length X protein was administrated using a canine viral vector (CAV2-X) in the *triceps surae* muscles (Figure 2A). CAV2-X passes through the neuromuscular junction and is transported retrogradely to MN somas in the lumbar spinal cord [19], where X protein is then expressed. Figure 2B shows the effective transduction of MNs on the L4 segment.

### 2.3. Effect of X Protein and PX3 Peptide on Motor Performance

**Figure 3:**
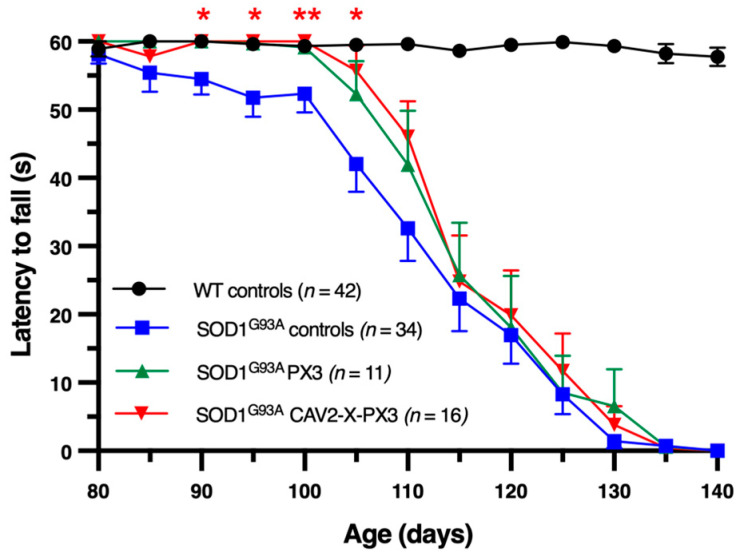
Delayed onset of motor symptoms in SOD1^G93A^-treated mice. After a training phase, the mice were tested from the age of 80 days. The test involved running on the cylinder of the rotarod rotating at a speed of eight revolutions per minute for 60 s. The test was carried out every 5 days. Each point represents the mean ± SEM. At each time point, the means were compared with a Kruskal–Wallis test followed by a post hoc Conover test. Red stars represent a significant difference between SOD1^G93A^ mice treated with the coadministration of X and PX3 and SOD1^G93A^ control mice (* *p* < 0.05; ** *p* < 0.01).

To evaluate the effect of treatments on both the onset and progression of motor deficits, we used a rotarod test from the age of 80 days (Figure 3). Control *SOD1^G93A^* animals presented motor deficits rapidly from the start of the test (blue curve) compared to WT mice (black curve, *** *p* < 0.001 at 90 days). At 100 days, 38% of the control *SOD1^G93A^* mice no longer passed the test. In contrast, 100% of the *SOD1^G93A^* mice treated with PX3 and CAV2-X-PX3 mice could run on the rotarod for 60 s. Moreover, until the age of 100 days, CAV2-X-PX3 mice presented no significant difference from WT mice.

The onset of motor deficits was observed in mice receiving both treatments at 105 days, when 18% and 12% of *SOD1^G93A^* mice treated with PX3 and CAV2-X-PX3, respectively, did not succeed in the test. At this time, CAV2-X-PX3 treatment significantly improved the motor performance of *SOD1^G93A^* mice. However, the improvement did not persist at or beyond 115 days. Altogether, these results showed that in treated *SOD1^G93A^* mice, the onset of motor deficits was delayed in the first part of the symptomatic stage.

### 2.4. Effect of X Protein and PX3 Peptide on Muscle Activity

To assess whether the observed improvement in motor performance was associated with the preservation of neuromuscular functionality, we studied the compound muscle action potential (CMAP) of the *Ts* at asymptomatic and symptomatic stages. This potential was recorded in response to supramaximal stimulation of the tibial nerve. The total recruitment of the *Ts* motor units was assessed by the maximum amplitude of the CMAPs.

**Figure 4:**
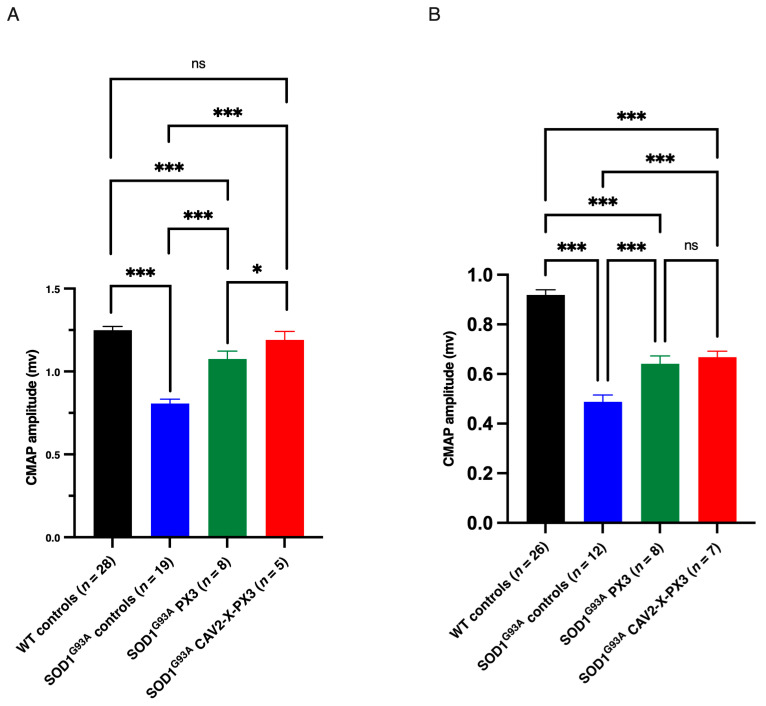
Increased maximum CMAP amplitude in SOD1^G93A^ mice treated with X protein and PX3 peptide in the presymptomatic and symptomatic stages. CMAPs developed by *Ts* muscles were recorded at 70 days (**A**) and 90 days (**B**) in response to supramaximal stimulation of the tibial nerve in WT control (black bar), SOD1^G93A^ control (blue bar), SOD1^G93A^ PX3 (green bar), and SOD1^G93A^X-PX3 (red bar) mice. The histogram bars correspond to the means ± SEMs. The data were analyzed with a nonparametric statistical test (Kruskal–Wallis) followed by a multiple comparison test (Dunn test), ns: not significant, * *p* < 0.05, *** *p* <0.001.

At both asymptomatic and symptomatic stages (70 and 90 days, respectively), control SOD1^G93A^ mice exhibited a significant decrease in CMAP amplitude compared to WT mice (blue bar, Figure 4).

In treated animals, the CMAPs of *SOD1^G93A^* mice treated with PX3 and *SOD1^G93A^* mice treated with CAV2-X-PX3 mice were significantly higher than those of *SOD1^G93A^* control mice at 70 and 90 days. Moreover, cotreated *SOD1^G93A^* mice exhibited a non-significantly different CMAP amplitude compared to WT animals at 70 days. These results showed a loss of motor units both at the asymptomatic and symptomatic stages in control *SOD1^G93A^* animals, while in treated animals, the motor units were partially preserved in the early stages of the disease.

During the symptomatic phase, although the CMAP amplitude decreased in CAV2-X-PX3 cotreated *SOD1^G93A^* mice, it was still significantly higher than that in control *SOD1^G93A^* animals, showing preservation of motor units at more advanced stages of the disease (90 days). Altogether, these results showed that in treated *SOD1^G93A^* mice, the motor units were preserved at the asymptomatic and symptomatic stages.

### 2.5. Effect of X Protein and PX3 Peptide on Motor Neuron Survival

**Figure 5:**
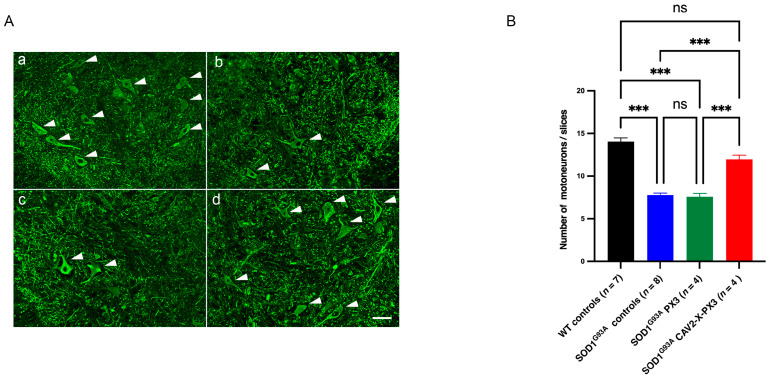
Increased survival of lumbar motor neurons in SOD1^G93A^ mice receiving cotreatment with X-PX3. (**A**): Lumbar spinal cord sections in the L4 segment in 90-day-old animals ((**a**): WT control mice; (**b**): SOD1^G93A^ control mice; (**c**): SOD1^G93A^ PX3 mice; (**d**): SOD1^G93A^ X-PX3 mice). The motor neurons were revealed by an anti-SMI32 antibody. Scale bar: 50 µm. (**B**): The number of motor neurons in lumbar segments L3 and L5 was determined. A total of 20 ventral horns per animal were analyzed. Each bar represents the average number of motor neurons. Error bars represent SEM. The data were analyzed with a Kruskal–Wallis test followed by a Dunn’s test (ns: not significant; *** *p* < 0.001).

MN degeneration is the main hallmark of ALS, and we determined the effect of X protein and PX3 peptide on the survival of lumbar MNs at 90 days of age. At this age, the number of lumbar MNs (SMI32 positive neurons) was significantly reduced in the control *SOD1^G93A^* mice compared to the control WT animals, and no significant difference was observed in the *SOD1^G93A^* PX3-treated mice (Figure 5). However, the *SOD1^G93A^* mice that received the co-treatment exhibited a significantly greater number of MNs compared to the untreated *SOD1^G93A^* mice.

These results show that despite the lack of a protective effect from the PX3 peptide alone, the co-administration of X protein and PX3 peptide increases the survival of lumbar MNs at the age of 90 days.

### 2.6. Effect of X Protein and PX3 Peptide on Weight and Lifespan

**Figure 6:**
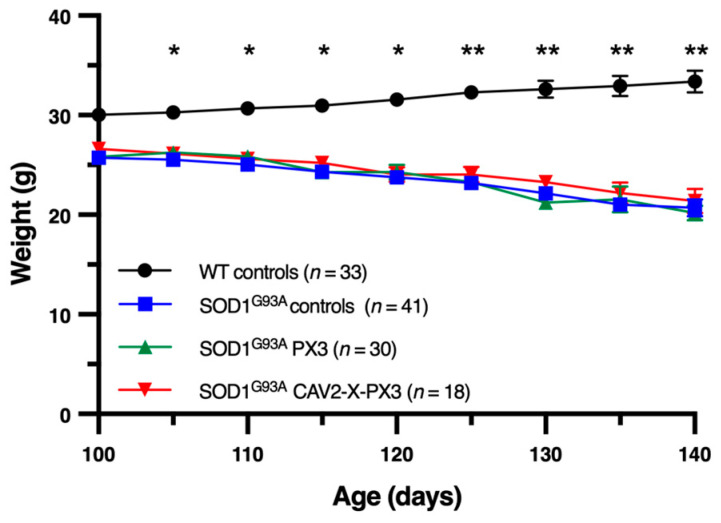
Body mass evolution between 100 days of age and death in SOD1^G93A^-treated mice. Mice were weighed every 5 days from 100 days of age until death. Each point represents the mean animal weight ± SEM. The means were compared with a two-way ANOVA test followed by a Tukey post hoc test. Significant differences were observed between WT controls (black rod symbols) and all groups of SOD mice; * *p* < 0.05; ** *p* < 0.01; no difference was revealed between controls (blue square symbols) and treated SOD mice (green and red triangle symbols). The disease end stage was defined as the time when the mouse could not right itself within 10 s when placed on its side.

Because progressive weight loss is an indicator of disease evolution, we evaluated this parameter daily from the age of 100 days until the death of the mice. As expected, although the WT animals continued to gain weight during this experimental window, we observed constant weight loss in treated *SOD1^G93A^* animals, with no significant difference between treated and untreated mice compared to WT mice (Figure 6).

**Figure 7:**
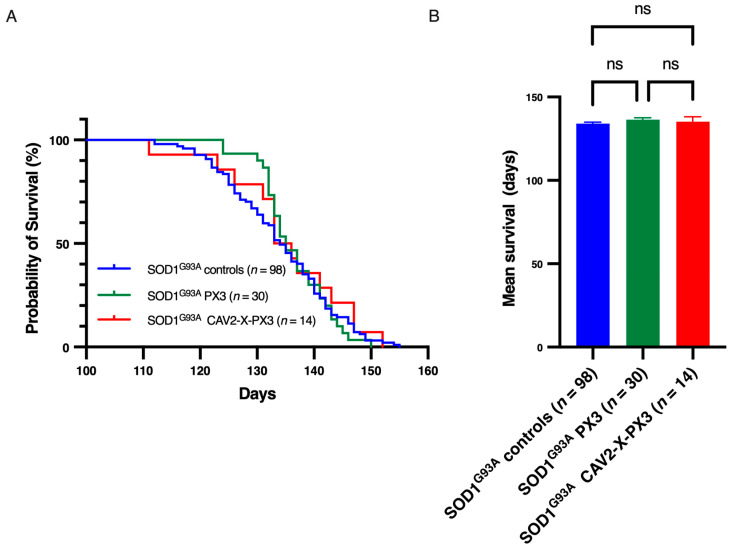
Life expectancy of SOD1^G93A^ mice was not improved by treatment with PX3 peptide alone or by the coadministration of PX3 peptide and X protein. (**A**): Kaplan–Meier survival plot of control SOD1^G93A^ mice (blue) and mice treated with PX3 (green) and X-PX3 (red). Curves were compared with a log rank (Mantel–Cox) test (*p* = 0.838). The median survival was 133 days for control mice, 136.5 days for PX3 mice, and 135.5 days for X-PX3 mice. (**B**): Mean age of survival in different treated groups. Each bar represents the mean ± SEM. The means were compared with one-way ANOVA (ns: not significant). The disease end stage was defined as the time when the mouse could not right itself within 10 s when placed on its side.

As animal survival is considered the primary endpoint of preclinical studies, the life expectancy of treated *SOD1^G93A^* mice was assessed across a large cohort. Figure 7A shows that the survival curves of control *SOD1^G93A^* mice, *SOD1^G93A^* mice treated with PX3, and *SOD1^G93A^* mice treated with CAV2-X-PX3 did not present any significant differences. The mean age of death of these three groups was 134 ± 0.9 days, 136.4 ± 1.1, and 135.2 ± 2.8 days, respectively (Figure 7B). Therefore, both treatments had no effect on the life expectancy of the animals.

## 3. Discussion

Our study clearly demonstrates the neuroprotective effect of X protein in a mouse model of ALS (*SOD1^G93A^* mice), with an obvious effect on motor performance and on both motor units and MNs survival. When the mice received a CAV2-X-PX3 treatment, they exhibited a delay in the onset of motor deficits that is associated with the preservation of motor units at 90 days of age, suggesting the increased bioavailability and selectivity of the action of X protein within the MNs. Moreover, our immunohistochemical study demonstrated that at this same age, a greater number of MNs survived. Despite the loss of MNs, which was comparable between the *SOD1^G93A^* animals treated with PX3 and the *SOD1^G93A^* control animals, the amplitude of CMAPs remained relatively preserved; this apparent discrepancy may be explained by some distal compensation mechanisms, such as axonal sprouting of preexisting MNs. Finally, the lifespan of the animals was not changed by double treatment in this aggressive genetic animal model. In accordance with previous publications, the results of this study suggest an effect on MNs in the initial period of the disease but less contribution in later disease progression [20,21].

Because the mitochondrial localization of X protein inhibits apoptosis in infected cells [16], the action of X protein at the mitochondrial level in our study could selectively block apoptosis via ROS-induced caspase apoptotic pathways [22]. Furthermore, the mitochondrial expression of X protein (or its derivative peptide PX3) is known to enhance filamentation of the mitochondrial network both under basal conditions and after oxidative stress [17]. It has been previously demonstrated that after treatment with X protein, mitochondrial membrane potential is restored, and the amount of ROS is decreased, consequently preventing neurons from being protected from neurodegeneration [17]. In fact, mitochondria (the main source of intracellular ROS) play a central role in ATP production (via oxidative phosphorylation), phospholipid biogenesis, calcium homeostasis, and apoptosis, so they are crucial for cell survival and metabolism [12]. Even almost 60 years ago, the ‘free radicals theory’ suggested a central role of the mitochondrion in normal mammalian aging; however, mitochondria play a more crucial role in the complex balance of cellular processes that may ultimately contribute to aging [23]. On the other hand, in addition to age-related comorbidities, mitochondrial dysfunction has been linked to various neurodegenerative disorders, including ALS, where structurally altered and aggregated mitochondria (with a swollen and vacuolated appearance) are one of the first pathological changes observed in the MNs of ALS patients [24,25], and similar alterations have been observed in the *SOD1^G93A^* model of ALS [26,27,28]. Therefore, it is evident that progressive modifications in mitochondrial morphology, energy production, and calcium dyshomeostasis are closely associated with the pathological process of ALS [12]. In this neurodegenerative disease, especially in the case of *SOD1^G93A^* mutation, the production of ROS is increased and leads to the production of apoptosis signals, inducing muscle deafferentation and the death of MNs in the spinal cord [20,29]. In our study, we showed that the administration of PX3 restored ATP levels in primary cultures of *SOD1^G93A^* MNs, clearly suggesting an impact of PX3 on the mitochondria localized inside MNs. However, although the coadministration of X protein and PX3 peptide delayed the onset of motor symptoms in *SOD1^G93A^* mice, this improvement was not maintained over time despite treatment prolongation. Moreover, the weight loss of the treated *SOD1^G93A^* mice was constant, with no difference from the untreated mice.

Despite these very encouraging results, we suggest that the insufficient neuroprotection observed (especially on life expectancy) could be because the expression of X protein was limited solely to the very few MNs innervating the *triceps surae* injected muscle. Moreover, the weak neuroprotective effect could be due to the limited diffusion of the peptide PX3 in the spinal cord after nasal instillation and because the derived peptide is small and easily degraded. Therefore, we believe that the administration protocol is not sufficiently optimized for the protein to be fully expressed in most MNs. This expression could be greatly enhanced (and more accurately targeted to MN mitochondria), and its therapeutic potential could consequently be improved. However, it is also probable that targeting only mitochondria is not sufficient to slow the progression of the disease. Currently, ALS is considered a multisystem degenerative disease, affecting not only MNs but also other neurons and explaining the wide clinical spectrum of the disease including cognitive disturbance (and frontotemporal degeneration) and other rare nonmotor symptoms such as anosmia, parkinsonism, sensory disturbance, or even supranuclear gaze palsy [30].

Moreover, initially, neurotoxicity in ALS was first considered as a sole cell autonomous process, meaning that damage within a selective population of affected neurons alone suffices to produce the disease. However, the selective expression of *mSOD1* in MNs failed to cause ALS-like disease in mice [31,32]. Nevertheless, the removal or reduction of *mSOD1* specifically in MNs extended the survival of mice by delaying disease onset and early disease progression [20,21,33] but had little effect on late disease progression. On the other hand, a growing body of evidence has shown that nonneuronal cells such as astrocytes, microglia, and oligodendrocytes may directly contribute to motor neuronal damage and cell death in ALS [34,35,36]. The removal of *mSOD1* in either microglia or astrocytes extends the survival of mice by slowing disease progression but does not delay the onset of the disease [20,37].

The combination of these results seems to indicate that individual cell types mediate different aspects of the disease and that the expression of *mSOD1* in both MNs and nonneuronal cells contributes to the ALS process in vivo. Finally, the pathophysiological complexity of ALS (including protein aggregation, glutamate excitotoxicity, oxidative stress, mitochondrial dysfunction, role of astrocytes/microglia and neuroinflammation, disturbed axoplasmic flow, etc.) explains the difficulties in finding a single treatment that is able to stop the disease process. It is likely that we should instead focus on therapeutic approaches that allow for the treatment of several targets in which mitochondria play a key role. Consequently, we need to extend the expression of X protein to all lower and upper MNs and to glial cells to validate the real therapeutic effect of PX. Therefore, the goal of our future work is to provide a broader expression of X protein on motoneurons and glial cells through a gene therapy strategy (more effective than the CAV2 vector or the PX3 peptide) such as an adeno-associated viral vector of serotype Rh10 that has proven its capacity to efficiently transduce the brain and the spinal cord [38].

## 4. Materials and Methods

### 4.1. Animals

Transgenic *SOD1^G93A^* mice Tg (SOD1-G93A)1Gur from Jackson Laboratory (Bar Harbor, USA) were maintained by breeding male heterozygous carriers with female (C57B6xSJL) F1 hybrids obtained from Charles River (*Janvier Labs*). Transgenic *SOD1^G93A^* mice and nontransgenic wild-type (WT) littermates were identified by genotyping for mutations in the human *SOD1* transgene using DNA extracted from pieces of the tail. For each animal, the genotype was also confirmed at the end of the study. Both male *SOD1^G93A^* mice and WT littermates were identified using a hand tattoo and kept in standard animal housing with free access to food and water. All experimental procedures were carried out according to the ‘Committee on Animal Health and Care of Bordeaux and the French Ministry of Higher Education, Research and Innovation’ (M.E.S.R.I.) (authorization number: A33-063-098).

Only male animals were examined in the present study to avoid ambiguity associated with reported sex-related differences in the mean survival time of *SOD1^G93A^* mice [39]. The male *SOD1^G93A^* mice became symptomatic at approximately 90 days of age. Symptoms included fine shaking, tremors, and spasticity in the legs [10]. Complete paralysis occurred within 30 to 40 days after the onset of symptoms, and the death of mice occurred at approximately 133 days. Body weight measurements were evaluated every day from 100 days of age until the end stage of the disease. The endpoint for experimental animals was established as the moment the mice were unable to right themselves within 10 s after being placed on their back. The animals were euthanized with an overdose of anesthetic (intraperitoneal injection of 300 mg/kg Exagon and 30 mg/kg Lidor).

### 4.2. Primary Culture of Mouse Motor Neurons

MNs were obtained from the dissociation of embryonic ventral spinal cords from 12.5-day *SOD1^G93A^* (ALS) and wild-type control littermate (heterozygous) mouse embryos (litters from heterozygous females coupled to homozygous *SOD1^G93A^* males), as described previously [13]. Briefly, the entire spinal cord was dissected and regrouped by genotype. The dorsal root ganglia and meninges were withdrawn as well as the dorsal part of the spinal cord. The remaining ventral spinal cords were chopped and dissociated with trypsin, and cells were separated by density gradient centrifugation with OptiPrep density gradient medium. The fractions containing MNs were carefully collected and filtered through a BSA cushion, and the purified MNs were seeded on poly-D-lysine- and laminin-coated culture dishes. Cultures were maintained in neurobasal medium supplemented with 2% B27, 2% inactivated horse serum, 100 U/mL penicillin, and 100 U/mL streptomycin and with glutamate, glutamine, and neurotrophic factors (GDNF 10 μg/mL, BDNF 1 μg/mL, CTNF 1 μg/mL) in 5% CO_2_ at 37 °C.

### 4.3. Measurement of Intracellular ATP

The intracellular ATP content of MNs was measured using a bioluminescent ATP determination kit (Molecular Probes, Life Technologies). A total of 20 microliters of a cell suspension of 5 × 10^5^ *SOD1^G93A^* or WT MNs/mL was plated in a 96-well plate. On DIV (‘days in vitro’) 1, cells were treated with PX3 (1 mg/mL) diluted at 1/200 for 48 h and then lysed using the lysis buffer provided with the kit (30 μL/well), and the lysates were immediately transferred into annotated tubes kept on ice and protected from light. The ATP concentration was determined by the light-emitting luciferase-catalyzed oxidation of luciferin with ATP, and bioluminescence was measured with a luminometer (*Luminoskan*, Labsystems, Finland). Standardization was performed using known quantities of standard ATP provided with the kit. Three independent cultures were performed for each condition.

### 4.4. Viral Vectors and Intramuscular Injection

The genes encoding X protein [16] were amplified by PCR and cloned and inserted into non-replicative canine adenovirus of type 2 (CAV2) vectors derived from the Manhattan strain. Genes were expressed under the control of the cytomegalovirus (CMV) promoter. CAV2-X constructs were generated and obtained as previously described [19]. Infectious titers were determined as ‘median tissue culture infectious doses’ (TCID50).

CAV2-X was injected into the two *triceps surae* muscles of each mouse. A single injection was performed early at 30 days of age. A total of 15 microliters of solution containing 2.5 × 10^10^ TCID50/mL CAV2-X was injected in three locations in the muscle (5 µL per injection) with a Hamilton syringe.

### 4.5. Synthesis of X-Derived Peptide PX3 and Intranasal Instillation

A cell-permeable peptide covering the C terminal parts (PX3, aa 59–87) of the BoDV-1-X protein (GenBank: ABW81015.1), coupled to an MPP sequence to ensure the targeting of the peptide to mitochondria, was synthesized with >99% purity (Genosphere Biotechnologies). The peptide solution (0.5 mg.mL^−1^) was applied using a pipette and tip. A quantity of 10 µL drops was placed into the nostrils and absorbed by the mouse five times per week from 30 days to death.

### 4.6. Rotarod

Before the test period, mice were trained every day for 10 days. Following the training period, motor tests were performed at 80 days until the mice were not able to stay on the rod. Every 5 days, the mice had to run at 8 rpm for 60 s. If the mice did not succeed, three consecutive trials were performed (with an interval of 15 min between each successive trial), and the longer time was recorded.

### 4.7. Electromyographic Study

The compound muscle action potential (CMAP) of the *triceps surae* (*Ts*) muscle of each animal was assessed at either 70 or 90 days of age. The mice were anesthetized by intraperitoneal injection of a ketamine/xylazine mixture (160 µL ketamine 0.5%, 80 µL xylazine 2%, 760 µL NaCL 9°/°°; 100 µL/10 g body weight). The sciatic nerve, visible under the biceps femoris, divides into three branches: the sural nerve (cutaneous nerve), the tibial nerve (nerve of the triceps surae), and the peroneal nerve (nerve of the calf extensors). The sural nerve is severed, and the tibial nerve is carefully separated from the peroneal nerve over an adequate length, after which the peroneal nerve is cut. This procedure isolates the tibial nerve, which innervates the triceps surae. A section of the sciatic nerve is then excised as close as possible to the spinal column, providing sufficient length for placement on the stimulation electrodes. Two stimulation electrodes were placed on this branch, and a supramaximal pulse was applied (*stimulator A.M.p.I. Master8*). The CMAP recordings were performed with two electrodes placed in the *triceps surae*. The electromyography (EMG) signal was amplified 1000 times and filtered with a bandpass of 0.3–5 kHz (*differential AC amplifier, A-M system*). CMAPs were sampled (5 kHz) using an A-D converter (*CED, Cambridge Electronic Design*, Cambridge, England). Interactive software (*Spike2*) was then used to manually determine CMAP amplitude. The liminal stimulation intensity was determined first, and sciatic nerve stimulation was progressively increased until the maximum EMG signal amplitude was reached. The total recruitment of motor units was evaluated by maximal CMAP amplitude.

### 4.8. Immunohistochemistry

At 90 days of age, mice were anesthetized with 300 µL of a mixture of Exagon (300 mg/kg) and Lidor (30 mg/kg) and were subjected to transcardial perfusion with 15 mL of ‘phosphate-buffered saline’ (PBS) containing 0.1% heparin. Then, the tissue was fixed with 4% paraformaldehyde (PFA) in PBS, and the lumbar segment of the spinal cord was harvested, postfixed for 2 h, and cryopreserved in 30% sucrose. The samples were individually embedded in *Tissue-Tek OCT* (Cellpath, Newport, Wales, UK) and frozen in isopentane at −60 °C. Transverse sections of 20 µm were cut with a cryotome (*Leica Cryostat CM1950S*, Wetzlar, Germany) between the L3 and L5 segmental levels. Sections were collected on Superfrost Plus Gold slides (Thermo Scientific, Waltham, MA, USA), permeabilized for 45 min at room temperature with 0.25% Triton X-100 in PBS, passed through washing buffered solutions, and blocked with 5% normal donkey serum and 3% BSA in PBS for 1 h at room temperature. Sections were then incubated overnight at 4 °C in a solution of 1× PBS, 5% normal donkey serum, and 3% BSA containing the primary antibodies mouse anti-SMI32 (1:500; *Eurogentec*, Seraing, Belgium) and/or rabbit anti-X (1:500, antibody provided by the D. Dunia team, Inserm, UMR1043, Toulouse, France). After washing, sections were incubated for 2 h at room temperature in 1:500 anti-mouse diluted secondary antibodies conjugated to Alexa Fluor 488 (*Invitrogen,* Thermo Scientific, Waltham, MA, USA) or ‘Alexa Fluor 594’ (*Eurogentec*) to reveal MNs and in 1:400 anti-rabbit diluted secondary antibodies conjugated with Alexa Fluor 488 (*Jackson* ImmunoResearch, Philadelphia, USA) to reveal cells that express X protein. Slides were then rinsed in PBS and mounted with a fluorescent medium with DAPI (*FluoProbes, Interchim, Montluçon, France*).

For quantification measurements, 10 sections of the lumbar segment were captured from each animal using a fluorescence microscope (*Nikon Inverted Microscope Eclipse Ti-U, Champigny sur Marne, France)* equipped with a digital camera (*Nikon DS-Qi2, Champigny sur Marne, France*) with a 10× objective. Quantification of MNs in the ventral horn was determined with *ImageJ* software (Version 1.54k). The data are presented as the mean ± SEM number of MNs by each ventral horn.

### 4.9. Statistical Analysis

All statistical analyses were performed with GraphPad Prism software (Version 10.4.0), R software (Version 4.3.3) [40] and the PMCMRplus Library [41]. The D’Agostino and Pearson normality test was performed on all datasets. Statistical differences between groups were determined using either a parametric (two-way ANOVA) or a non-parametric test (the Kruskal–Wallis test) when appropriate. For the survival analysis, Kaplan–Meier curves and log-rank tests (Mantel–Cox) were employed. Body weight evaluation was analyzed using a two-way ANOVA followed by a post hoc Tukey test. Rotarod, EMG, and quantitative analyses of MN survival data were analyzed with the Kruskal–Wallis test followed by a multiple comparison Dunn test or Conover test. The level of statistical significance was 95% (*p* < 0.05).

## Data Availability

The original contributions presented in this study are included in this article; further inquiries can be directed to the corresponding authors.
